# Supplementary material for: Content-rich biological network constructed by mining PubMed abstracts
Source: BMC Bioinformatics. 2004 Oct 8;5:147. doi: 10.1186/1471-2105-5-147 (PMC528731; doi:10.1186/1471-2105-5-147)
Supplement: Additional File 2 — The original results of the above study (non-essential files are deleted to keep the file size under the limit set by BMC bioinformatics). [file 1471-2105-5-147-S2.bz2 › chilibotAdditionalFile2/dip05/485ID9312096E10580/html/BLR1_CCL4.html]

 


 **BLR1** and **CCL4** 
  
Found 2 abstracts in PubMed, retrieved 2.  
 

 What does Google say? 
 PDF only 
| .edu only 

---

**Interactive relationship** (e.g. stimulation, inhibition, etc)

**Neutral relationship**- **BLR1**  shows significant relationship to receptors for chemokines IL 8, MIP 1 beta  [ **CCL4** ]  and neuropeptides.  Ref: 7920182 Cell Mol Biol Noisy le grand , 1994
